# Inflammatory mediators drive neuroinflammation in autism spectrum disorder and cerebral palsy

**DOI:** 10.1038/s41598-023-49902-8

**Published:** 2023-12-18

**Authors:** Uyen Thi Trang Than, Liem Thanh Nguyen, Phuong Hoang Nguyen, Xuan-Hung Nguyen, Dong Phuong Trinh, Diem Huong Hoang, Phuong Anh Thi Nguyen, Van Duc Dang

**Affiliations:** 1 Vinmec Hi-Tech Center and Vinmec-VinUni Institute of Immunology, Vinmec Healthcare System, Hanoi, 100000 Vietnam; 2 Vinmec Research Institute of Stem Cell and Gene Technology, Vinmec Healthcare System, Hanoi, 100000 Vietnam; 3 College of Health Sciences, VinUniversity, Hanoi, 100000 Vietnam; 4 Faculty of Biology, VNU University of Science, Vietnam National University, Hanoi, Vietnam; 5 Vinmec International Hospital Times City, Vinmec Healthcare System, Hanoi, 100000 Vietnam; 6 Deutsches Rheuma-Forschungszentrum Berlin, Leibniz Institute, Charitéplatz 1, 10117 Berlin, Germany

**Keywords:** Neuroimmunology, Neuroimmunology

## Abstract

Inflammation conditions are associated with autism spectrum disorder (ASD) and cerebral palsy (CP), primarily observed in the peripheral immune system. However, the extent of neuro-inflammation and neuro-immune dysregulation remains poorly studied. In this study, we analyzed the composition of cerebrospinal fluid (CSF) to uncover the inflammatory mediators driving the neuro-immune system in ASD and CP patients. Our findings revealed that ASD patients had elevated levels of four inflammatory cytokines (TNF-α, IL-4, IL-21, and BAFF) compared to controls, while CP patients exhibited increased levels of eight inflammatory cytokines (IFN-γ, GM-CSF, TNF-α, IL-2, IL-4, IL-6, IL-17A and IL-12), one anti-inflammatory cytokine (IL-10), and five growth factors (GFs) (NGF-β, EGF, GDF-15, G-CSF and BMP-9) compared to both controls and ASD patients. Additionally, intrathecal infusion of autologous bone marrow mononuclear cells (BMMNCs) led to a slight decrease in TGF-β and GDF-15 levels in the CSF of ASD and CP patients, respectively. Our study provides new insights into the molecular composition of CSF in ASD and CP patients, with the potential to develop more effective diagnosis methods and improved treatment for these diseases.

*Clinical trial registration* CSF samples used in this study are from clinical trials NCT03225651, NCT05307536, NCT02569775, NCT03123562, NCT02574923, NCT05472428 and previous reports [7, 9, 17–19].

## Introduction

ASD is a neurodevelopmental condition affecting verbal communication, social interaction, and behaviors^1^. CP, the most common childhood disability, impacts movement, posture, and coordination^2^. While the etiology of ASD is unclear and the symptoms as well as severity vary among individuals, CP results from brain damage during infancy, preterm birth or birth asphyxia^1,2^. Recent reports indicate an association of ASD and CP with immunological dysfunction. Both conditions have been linked to increased levels of inflammatory cytokines, including IL-1β, IL-8, IL-6, and TNF-α in biological fluids^3–6^.

Current treatments for ASD aim to maximize a child's capabilities, enhance daily functioning, and support their overall quality of life^1^. In CP, there are various treatment approaches, including medications, physiotherapy, occupational therapy, surgical procedures, and other interventions tailored to the specific symptoms and affected body areas^2^. Nonetheless, there remains a gap in therapeutic treatments that effectively address the core symptoms of ASD and CP, prompting the exploration of innovative therapies. Cell therapies, specifically BMMNC infusions, show promise in improving symptoms and quality of life in both conditions^7–10^. However, the underlying mechanisms of these therapies require further exploration.

The CSF, which surrounds the brain and spinal cord, plays a critical role in maintaining the central nervous system (CNS) environment^11–13^. It can also serve as an indicator of neuro-inflammation in various neurological disorders^14–16^. Analyzing the molecular composition of CSF, particularly cytokines, can provide insights into the pathophysiology of ASD and CP. This analysis can also aid in subtype classification, disease severity assessment, diagnosis, and treatment development.

In this study, we examined 28 molecules, including inflammatory cytokines, anti-inflammatory cytokines, and growth factors, in CSF samples from ASD, CP, and control patients. We also compared these molecules in ASD and CP patients at pre- and post-cell therapy to gain insight into the treatment's impact.

## Results

### Cohorts and patient characteristics

After obtaining written informed consent, we collected CSF samples from patients with ASD, CP, and SB, which would have otherwise been discarded as part of clinical trials NCT03225651, NCT05307536, NCT02569775, NCT03123562, NCT02574923, NCT05472428 and previous reports^7,9,17–19^. In total, we obtained 115 CSF samples from 26 patients with ASD, 28 patients with CP, and eight patients with SB in which 26 ASD and 27 CP patients provided samples at two time points: before the first BMMNC infusion (baseline) and five to 12 months later (before the second BMMNC infusion). Demographic details are summarized in Table 1.

**Table 1:** Patient characteristics.

	ASD	CP	SB
Total numbers	26	28	8
Age Range (year) Mean (± SD)	3–11 6.5 (± 2.0)	1–16 5.4 (± 3.9)	1–18 6.2 (± 4.8)
Interval time of 1st and 2nd CSF collection (month)	5–12	5–12	
CSF volume (mL) Mean (± SD)	3.64 ± 0.66* 4.1 ± 0.57^#^	3.12 ± 0.35* 3.57 ± 0.66^#^	4.1 ± 1.96*

Data were presented as Mean (± SD).

*First CSF collection (before the first BMMNC infusion).

^#^Second CSF collection (5–12 months after the first BMMNC infusion − before the second BMMNC infusion).

### Soluble proteins are detectable in CSF

To identify the soluble protein composition in the CSF of patients with ASD, CP, and SB at baseline, we conducted a comprehensive analysis of 28 molecules (Table 2). Our focus was on cytokines and GFs that have been previously reported to exhibit significant changes in the biological liquids of patients with ASD and CP (Supplemental Table 1). We also included additional molecules based on our predictions regarding their potential involvement in driving the disease progression (Supplemental Table 1).

**Table 2:** Concentration of soluble proteins detected in CSF samples from ASD, CP, and SB patients at baseline.

Cytokines/growth factors	ASD (pg/mL) Mean ± SD (Detected/total samples)	CP (pg/mL) Mean ± SD (Detected/total samples)	SB (Control) (pg/mL) Mean ± SD (Detected/total samples)
APRIL	9370 ± 102 (26/26)	747 ± 242 (28/28)	9320 ± 86.5 (8/8)
BAFF	7.61 ± 2.1 (26/26)	7.17 ± 1.65 (28/28)	5.78 ± 2.38 (8/8)
BDNF	Not detectable	Not detectable	Not detectable
BMP-9	15.2 ± 4.32 (26/26)	36.1 ± 14.7 (27/28)	11.7 ± 4.48 (6/8)
EGF	8.55 ± 2.34 (26/26)	21.40 ± 6.70 (28/28)	7.77 ± 2.24 (7/8)
FGF-2	276 ± 5.2 (3/20)	267 ± 6.0 (3/20)	Not analyzed
G-CSF	116 ± 23.4 (26/26)	233 ± 51.8 (28/28)	93.3 ± 40.3 (8/8)
GDF-15	117 ± 35.6 (26/26)	183 ± 141 (28/28)	73.8 ± 30.9 (8/8)
GM-CSF	127 ± 24.5 (26/26)	224 ± 41.1 (28/28)	122 ± 32.2 (7/8)
HGF	801 ± 259 (20/20)	707 ± 221 (20/20)	Not analyzed
IFN-γ	14.3 ± 9.58 (22/26)	43.6 ± 11.9 (28/28)	12.6 ± 7.24 (5/8)
IL-1β	13 ± 3.84 (26/26)	13.9 ± 4.71 (28/28)	11.4 ± 2.26 (6/8)
IL-2	36 ± 6.28 (26/26)	82.6 ± 19 (28/28)	37.3 ± 6.22 (6/8)
IL-4	62.5 ± 16.7 (26/26)	83.7 ± 21.4 (28/28)	44.2 ± 15.6 (8/8)
IL-5	187 ± 165 (25/26)	70.4 ± 36.4 (27/28)	89. 9 ± 129 (8/8)
IL-6	78.8 ± 18.4 (26/26)	160 ± 42.9 (28/28)	65 ± 14.5 (6/8)
IL-10	3.34 ± 0.835 (26/26)	6.83 ± 2.62 (28/28)	3.36 ± 1.57 (7/8)
IL-12p70	40.1 ± 15.6 (26/26)	52.1 ± 16.2 (28/28)	33.8 ± 11 (6/8)
IL-17A	47.6 ± 13.9 (26/26)	63 ± 20.4 (28/28)	46 ± 18.1 (7/8)
IL-21	73.2 ± 33.6 (26/26)	61.5 ± 26.2 (28/28)	45.6 ± 34.4 (7/8)
IL-31	35.8 ± 10.9 (26/26)	27.8 ± 5.25 (28/28)	30.2 ± 13 (7/8)
IL-33R	166 ± 23.9 (26/26)	215 ± 118 (23/28)	156 ± 32.6 (8/8)
M-CSF	18.8 ± 8.99 (22/26)	73.1 ± 68.7 (10/28)	21.8 ± 12.3 (2/8)
NGF-β	11.3 ± 2.8 (26/26)	13 ± 2.25 (28/28)	8.3 ± 2.58 (7/8)
PDGF	55.4 ± 42.7 (20/20)	51.9 ± 17.7 (20/20)	Not analyzed
TGF-β	23 ± 25.5 (10/20)	5.31 ± 0.6 (2/20)	Not analyzed
TNF-α	107 ± 29.7 (26/26)	133 ± 52.5 (28/28)	73.8 ± 38.7 (7/8)
VEGF	84.5 ± 26 (20/20)	91.2 ± 55.2 (20/20)	Not analyzed

Table shows the concentration of soluble proteins and the number of detected samples/total analyzed samples for each soluble protein from CSF samples of ASD, CP, and SB patients at baseline using Luminex assay. Data were presented as Mean ± SD.

**Figure 1:**
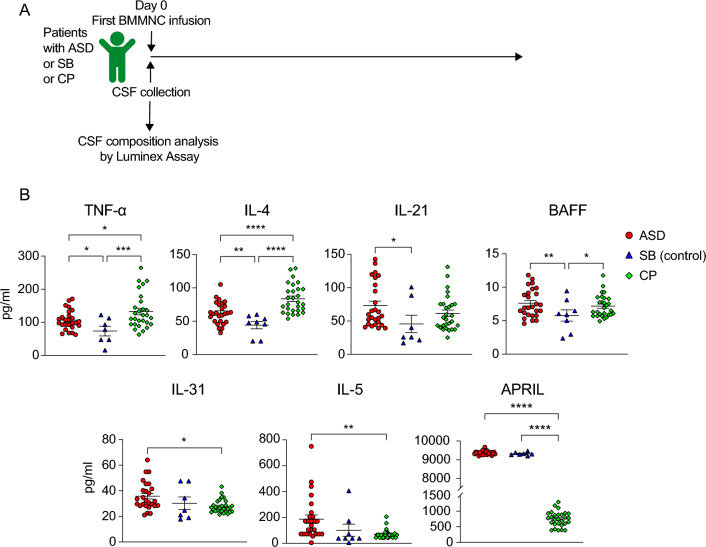
Elevated levels of inflammatory cytokines in CSF from ASD patients. (**A**) Experiment design. CSF samples were collected at baseline (before the first autologous BMMNC infusion) for CSF composition analysis by Luminex assay. (**B**) Graphs show the concentration of indicated inflammatory cytokines quantified by Luminex assay from CSF samples of ASD compared to SB and CP patients. Only cytokines significantly increased in the CSF samples from ASD patients compared with SB or CP were shown. Only samples in which cytokine concentration was detected by Luminex assay as indicated in Table 2, were included in the analysis. Groups were compared using ANOVA and Turkey HSD tests. Data were presented as Mean ± SEM for the concentration of indicated cytokines. Considered *p*-values were: *p < 0.05; **p < 0.01; *** *p* < 0.001; **** *p* < 0.0001. *p* values > 0.05 are not shown.

We quantified these 28 molecules in the CSF samples using Luminex assay (Fig. 1A) as this method enables the simultaneous measurement of multiple molecules using only 25–50 µL of the sample. While 20 molecules were quantified by all analyzed samples (Table 2), we could only detect M-CSF, IL-5, IL-33R, BMP-9, IFN-γ, FGF-2, and TGF-β in most or some samples. Particularly, M-CSF was detected in 22 out of 26 ASD samples, 10 out of 28 CP samples, and two out of eight SB samples. IL-5 was detectable in 25 out of 26 ASD samples and 27 out of 28 CP samples. IL-33R was detectable in 23 out of 28 CP samples. BMP-9 was detectable in 27 out of 28 CP samples and six out of eight SB samples, and IFN-γ was detectable in 22 out of 26 ASD samples and five out of eight CP samples (Table 2). Additionally, FGF-2 was detected in only three out of 20 samples from both ASD and CP patients. TGF-β was detected in only ten of 20 CSF samples from ASD patients and two out of 20 CSF samples from CP patients (Table 2). Unexpectedly, BDNF was not detected in any CSF samples from all analyzed patients (Table 2). We conclude that soluble proteins in CSF samples can be quantified using Luminex assay.

### TNF-α, IL-4, IL-21, BAFF, IL-31, IL-5, and APRIL are increased in CSF from ASD patients

The detection of soluble proteins in CSF samples prompted us to investigate potential differences in the levels of these proteins among three patient cohorts at baseline (Fig. 1A). Given their potential reflection of neuro-inflammatory conditions and disease pathogenesis, we specifically focused on inflammatory cytokines. In live with previous findings that reported increased levels of serum TNF-α^20,21^, IL-4^22^, and IL-21 by peripheral blood mononuclear cells in ASD patients^23^ (Supplementary table 1), herein we found marked elevations of TNF-α, IL-4, and IL-21 in the CSF samples from ASD compared with the SB patients which is likely a non-neuroinflammatory disease (Fig. 1B). Additionally, the CSF from ASD patients displayed increased levels of IL-31 and IL-5 compared to CP patients (Fig. 1B). Considering the increased levels of serum IgG, IgG2 and IgG4 antibodies^24^ and the presence of autoantibodies recognizing brain antigens in ASD^25–27^, we therefore examined whether BAFF and APRIL, which are required for maintenance and survival of antibody-secreting cells^28^, were present in the CSF from ASD patients. Remarkably, BAFF and APRIL were found to have increased levels in the CSF samples from ASD compared to SB and CP patients, respectively (Fig. 1B). Altogether, our data indicate elevated contents of inflammatory cytokines TNF-α, IL-4, IL-21, BAFF, IL-31, IL-5 and APRIL in CSF samples from ASD patients, suggesting the potential inflammatory conditions and immune dysfunctions that might contribute the pathogenesis and progression of this disease.

### IFN-γ, GM-CSF, TNF-α, IL-2, IL-4, BAFF, IL-6, IL-17A, IL-12p70, and IL-10 are increased in CSF from CP patients

**Figure 2:**
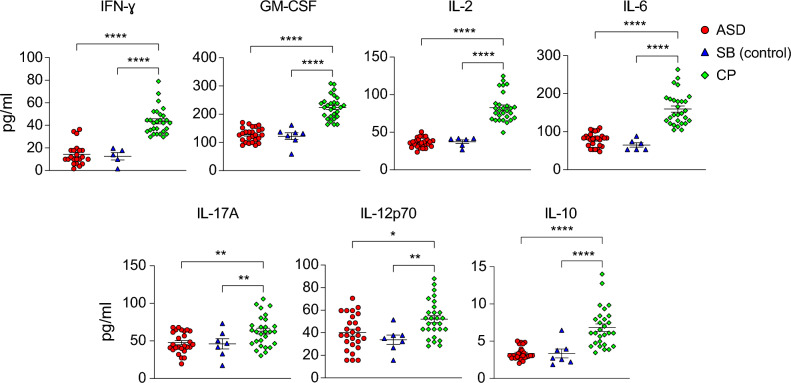
Elevated levels of inflammatory cytokines in CSF from CP patients. Graphs show the concentration of indicated inflammatory cytokines, including IFN-γ, GM-CSF, IL-2, IL-6, IL-17A, and IL-12, in the CSF samples from CP patients compared to those from ASD and SB patients. Only cytokines significantly increased in the CSF samples from CP patients compared with those from ASD and SB were shown. Only samples in which cytokine concentration was detected by Luminex assay as indicated in Table 2, were included in the analysis. Groups were compared using ANOVA and Turkey HSD tests. Data were presented as Mean ± SEM for the concentration of indicated cytokines. Considered *p*-values were: *p < 0.05; **p < 0.01; **** *p* < 0.0001. *p* values > 0.05 are not shown.

Next, we examined the abnormal levels of inflammatory cytokines present in the CSF of CP compared to SB controls. In addition to TNF-α, IL-4, and BAFF, as shown in Fig. 1, we observed elevated levels of other inflammatory cytokines, including IFN-γ, GM-CSF, IL-2, IL-6, IL-17A, and IL-12 in CSF samples from CP patients compared to those from SB and ASD patients (Fig. 2). These findings indicate severe inflammatory conditions and neuro-immune dysregulations in CP. We conclude that the CSF samples from CP patients exhibit elevated levels of inflammatory cytokines IFN-γ, GM-CSF, TNF-α, IL-2, IL-4, IL-6, IL-17A, and IL-12. Next, we determined the level of anti-inflammatory cytokines in the CSF samples from three cohorts and found that the level of IL-10 was higher in the CSF samples from CP patients compared with SB and ASD (Fig. 2). This clearly indicates the imbalance between inflammatory and anti-inflammatory cytokines in the CNS of the CP patients.

### NGF-β, EGF, GDF-15, G-CSF and BMP-9 are increased in CSF from CP patients

**Figure 3:**
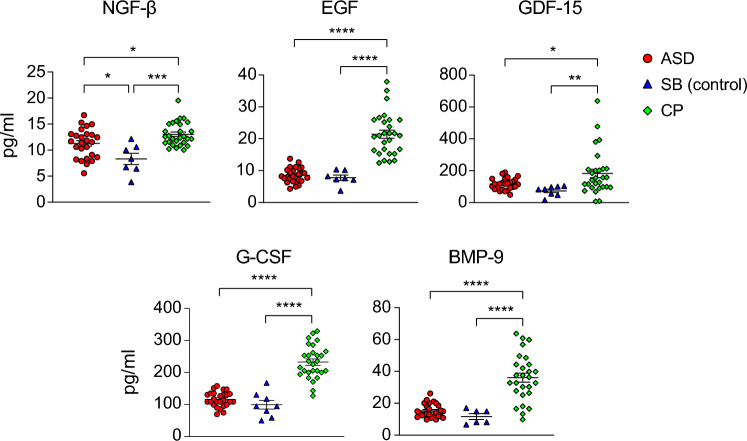
Increased levels of growth factors in CSF from CP patients. Graphs show the concentration of indicated growth factors, including NGF-β, EGF, GDF-15, G-CSF, and BMP-9, in the CSF samples from CP compared to ASD and SB patients. Only growth factors significantly increased in the CSF samples from CP patients compared with those from ASD and SB were shown. Only samples in which cytokine concentration was detected by Luminex assay as indicated in Table 2, were included in the analysis. Groups were compared using ANOVA and Turkey HSD tests. Data were presented as Mean ± SEM for the concentration of indicated cytokines. Considered *p*-values were: *p < 0.05; **p < 0.01; *** *p* < 0.001; **** *p* < 0.0001. *p* values > 0.05 are not shown.

The CNS, encompassing the brain and spinal cord, plays a central role in controlling various body functions, especially sensory and motor functions. The development and function of the CNS are intricately regulated by numerous brain GFs. Given the previous studies highlighted the association of serum GFs with ASD and CP (Supplemental Table 1), we sought to investigate whether brain GFs are impaired in the CSF samples of these patients. We found significant elevations of NGF-β, EGF, GDF-15, G-CSF, and BMP-9 in the CSF from CP patients compared with the other groups (Fig. 3), while similar amounts were detected between ASD and SB, except NGF-β that was higher in ASD compared to SB (Fig. 3). Additionally, we did not detect any significant difference in other tested GFs, including M-CSF, FGF-2, PDGF-BB, and VEGF between CP and ASD (Table 2 and data not shown).

### Decrease TGF-β in CSF of ASD patients and GDF-15 in CSF of CP patients after autologous BMMNC infusion therapy

**Figure 4:**
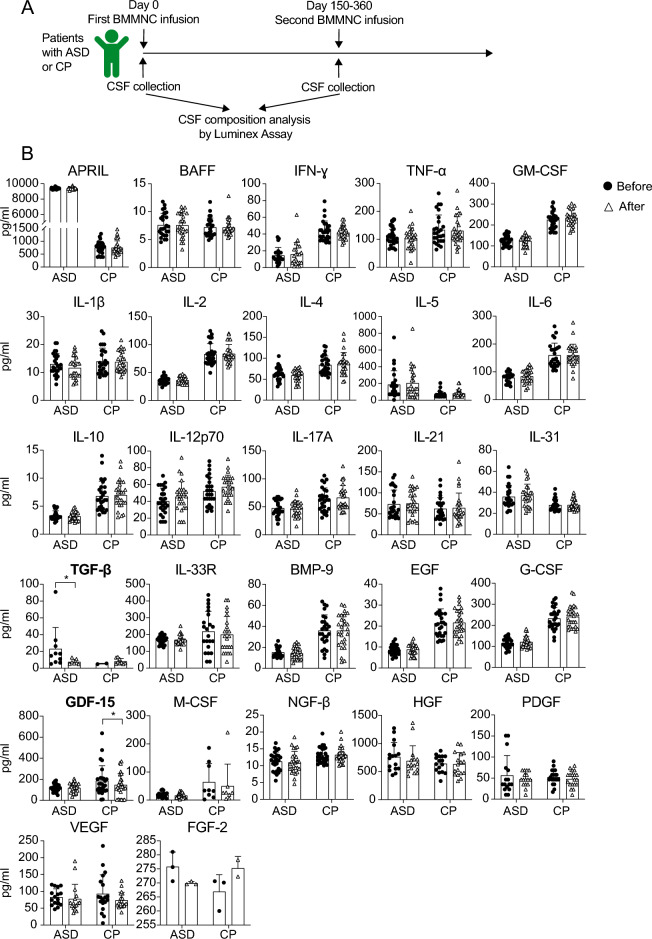
Changes in cytokines and growth factors in CSF from ASD and CP patients after autologous BMMNC infusion. (**A**) Experiment design. CSF samples were collected from ASD and CP patients at day 0 as the baseline (before the first BMMNC infusion) and at day 150–360 (after the first BMMNC infusion and before the second BMMNC infusion) for CSF composition analysis by Luminex assay. (**B**) Graphs show the concentration of 27 analyzed molecules in the CSF from ASD and CP patients at baseline compared with those at 150–360 days after BMMNC infusion. Only samples in which cytokine concentration was detected by Luminex assay were included in the analysis. Groups were compared using paired T-test or Wilcoxon Signed Rank Test. Data were presented as Mean ± SEM for the concentration of indicated cytokines. Considered *p*-values were: *p < 0.05. *p* values > 0.05 are not shown.

Previous studies have reported the safety and tolerability of BMMNC infusion in children with ASD and CP^7,9,17,18^. Furthermore, this therapeutic approach has demonstrated efficacy in enhancing gross motor function and muscle tone in children with CP^9^. To access the impact of intrathecal infusion of autologous BMMNCs on the levels of cytokines and GFs in the CSF of ASD and CP patients and explore the potential of these molecular markers in evaluating therapy efficacy, we quantified 28 cytokines and GFs in CSF samples obtained before and five to 12 months after the BMMNC infusion therapy (Fig. 4A). Our analysis found a reduction of TGF-β and GDF-15 in the CSF samples from ASD and CP patients, respectively. However, the remaining analyzed molecules did not exhibit significant changes in their levels at five to 12 months post-BMMNC infusion therapy in both ASD and CP cohorts (Fig. 4B). We conclude that autologous BMMNC infusion appears to induce a slight decrease in TGF-β and GDF-15 levels in the CSF of patients with ASD and CP, respectively.

## Discussion

The persistent and excessive cytokine production is a hallmark of inflammation, driving the pathogenesis and progression of chronic inflammatory diseases^29^. Therefore, cytokine profiling can reveal the status of inflammation and immune system dysregulation, offering therapeutic targets for treating cytokine-mediated conditions. In this study, we quantified 28 molecules and observed the increased levels of several key inflammatory cytokines in CSF samples from ASD and CP patients, indicating the neuro-inflammation in CNS of patients with ASD and CP.

TNF-α, BAFF, IL-4, and IL-5 have been found at increased levels in the sera of patients with ASD compared to healthy controls, and these changes are associated with ASD severity^3,20,22,30–32^ (Supplemental Table 1). Additionally, elevated concentrations of IL-4 and IL-5 were observed in the sera of women at 15–19 weeks of gestation who later gave birth to a child diagnosed with ASD compared to women who gave birth to normal children^33^ (Supplemental Table 1). Herein, we observed increased levels of TNF-α, BAFF, IL-4, and IL-5 in the CSF of patients with ASD compared to SB patients, suggesting a correlation between serum and CSF cytokine levels that contribute to ASD pathophysiology. In addition, the markedly increased levels of BAFF, IL-4, IL-5, and IL-21 in the CSF of children with ASD suggest their potential involvement in disease pathogenesis through humoral immunity responses. This hypothesis aligns with the role of BAFF, IL-4, IL-5, and IL-21 in B cell maturation, proliferation, and antibody-secreting cell differentiation^34–38^. Notably, patients with ASD display increased amounts of serum IgG, IgG2, and IgG4 antibodies. These data strongly support the hypothesis that ASD might be an antibody-mediated autoimmune disease^24^. Indeed, serum anti-myelin basic protein autoantibodies significantly elevated in ASD compared to healthy controls^27^. In other contexts, IL-4, IL-5, and IL-21 can act as anti-inflammatory cytokines in regulatory mechanisms mediated by regulatory T and B cells^39–41^. Therefore, we do not exclude the possibility that the increased levels of IL-4 and IL-5 in the CSF of patients with ASD might be the consequence of a negative feedback response to the elevation of other inflammatory cytokines in ASD patients. In this case, IL-4 and IL-5 could be classified as anti-inflammatory cytokines.

Recent studies highlighted increased levels of IL-1β, TNF-α, IL-6, IL-8, and IL-10 in the saliva of CP patients^42^ (Supplemental Table 1). In our study, we found a significant increase in the levels of TNF-α, IL-6, and IL-10, along with IFN-γ, GM-CSF, IL-2, IL-4, IL-17A, BAFF, and IL-12p70 in the CSF of CP patients. Additionally, IL-1β, IL-6, and TNF-α, which were detected in the CSF samples from CP patients in this study, were also reported to be at higher levels in saliva and maternal amniotic fluids of CP patients^42,43^.

The diagnosis of ASD and CP is typically established according to the Diagnostic and Statistical Manual of Mental Disorders, Fifth Edition^44^, and Gross Motor Function Classification System^45^, respectively. Nevertheless, the need for new biomarkers persists since the symptoms and severity of ASD and CP are not the same in all cases. CSF has emerged as a primary target for biomarker exploration due to its proximity to the CNS, offering insights into pathogenesis, diagnosis, response to drug treatment, and eventually the development of improved treatments for ASD and CP. Our findings suggest that a set of four cytokines (TNF-α, BAFF, IL-4, and IL-5) and a set of ten cytokines (IFN-γ, GM-CSF, TNF-α, IL-2, IL-4, IL-6, IL-17A, BAFF, IL-12p70, and IL-10) could serve as potential biomarkers for ASD and CP, respectively. Additionally, numerous efforts have been directed towards identifying blood-based biomarkers for ASD due to its greater accessibility compared to CSF^3,20,22,30–32,42^. The consistency of TNF-α, BAFF, IL-4, and IL-5 in the CSF of patients with ASD and TNF-α, IL-6, and IL-10 in the CSF of patients with CP in our study, with previous reports on their presence in the serum of ASD and CP patients^3,20,22,30–32,42^, suggests the feasibility of determining these cytokines from the blood of patients with ASD and CP for early diagnosis. This could help avoid unnecessary lumbar punctures, particularly in small children, given the invasive nature of the procedure. Nonetheless, this option might be considered in select cases where blood samples do not provide sufficient information (e.g., for evaluating intrathecal stem cell/drug infusion therapies).

Cytokine-mediated inflammatory responses drive key processes of chronic inflammatory diseases. In our study, we observed an elevation of TNF-α in both ASD and CP, suggesting a shared mechanism involving TNF-α in both diseases. Despite its well-established role in various homeostatic and inflammatory processes, the specific contributions of TNF-α to ASD and CP remain largely unexplored. Notably, TNF-α is a key inflammatory cytokine implicated in autoimmune inflammatory diseases of the CNS^46^. In the CNS, TNF-α is produced by endothelial cells, neurons, and infiltrating immune cells. Additionally, infiltrating T cells can stimulate the local production of TNF-α by microglial cells through the secretion of IFN-γ. Elevated TNF-α levels can trigger a cascade of events, including excitotoxic cell death in neurons, modulation of glutamate release and re-uptake in astrocytes, activation of microglial cells, disruption of the blood–brain barrier, and the infiltration of inflammatory immune cells and molecules into the CNS^47^. These mechanisms may also be relevant in ASD, as there are reports indicating altered expression of genes associated with the blood–brain barrier in ASD, correlating with increased neuroinflammation in this disorder^48^. Given the increased levels of TNF-α in both ASD and CP, it emerges as a potential therapeutic target. Anti-TNF-α therapy, approved for various chronic inflammatory diseases and CNS autoimmune conditions^47^, might be explored for alleviating symptoms and slowing down disease progression in ASD and CP using TNF-α inhibitors. While TNF-α stands out, we acknowledge the possibility that other cytokines, such as BAFF, IL-4, and IL-5 for ASD, and IFN-γ, GM-CSF, IL-2, IL-4, IL-6, IL-17A, BAFF, and IL-12p70, for CP, may also contribute to the pathophysiology of these disorders and might serve as therapeutic targets.

The absence of CSF samples from healthy children poses a challenge in establishing an ideal control group for comparing cytokine and GF profiles with those from ASD and CP patients. Ethical constraints prevent the acquisition of CSF samples from healthy individuals due to the invasive nature of lumbar punctures, especially in children without existing health concerns. Consequently, our comparative analysis focused on assessing these molecules in CSF of ASD patients relative to SB and CP patients. As SB patients have some neurological impairment, it is possible that certain tested molecules could be potentially elevated in the CSF of SB compared to real healthy donors. Remarkably, the majority of cytokines that displayed elevated levels in the CSF of ASD or CP compared with SB in our study exhibited a consistent pattern of increase in the blood of ASD or CP compared to healthy donors, as documented in previous reports (Supplemental Table 1).

Following BMMNC infusion therapy, we observed a decrease only in the levels of TGF-β and GDF-15 in CSF samples from ASD and CP, respectively (Fig. 4). This might be because BMMNC infusion led to immediate changes in CSF cytokine levels, but the collection of CSF samples was performed at a very late time point (five to 12 months post-therapy), thus not adequately reflecting the efficacy of this cell therapy. Another possibility is that the 28 tested molecules might not encompass all factors responding to this therapy. Therefore, a more extensive panel with additional cytokines and GFs should be explored for a more comprehensive understanding of the impact of this cell therapy.

In conclusion, our findings unequivocally reveal elevated levels of inflammatory cytokines in the CSF of the patients with ASD and CP. Specifically, we have identified a set of seven inflammatory cytokines, including TNF-α, IL-4, IL-21, BAFF, IL-31, IL-5, and APRIL, were increased in the CSF of the patients with ASD, and a set of ten cytokines, including IFN-γ, GM-CSF, TNF-α, IL-2, IL-4, IL-6, IL-17A, BAFF, IL-12p70 and IL-10, were increased in the CSF of the patients with CP. These findings provide a novel insight into the molecular pathophysiology of these diseases. Moreover, these cytokines hold potential as therapeutic targets to restore the balance between inflammation and anti-inflammation, thereby re-establishing immune regulation mechanisms in these diseases. Further investigation is necessary to elucidate the involvement of these molecules in the pathogenesis and progression of ASD and CP and assess their potential as biomarkers for disease severity. Notably, the autologous BMMNC infusion, a new therapy for ASD and CP, showed no significant alterations in the levels of the analyzed molecules, except for a decrease in TGF-β in ASD and GDF-15 in CP. These data suggest that these two molecules might mediate the effects of BMMNC infusion therapy in these diseases.

## Materials and methods

### Study design

The study aimed to assess and quantify the CSF compositions of patients with ASD, CP and SB, and to investigate whether autologous BMMNC infusion therapy has any impact on the CSF compositions of patients with these diseases.

### Inclusion and exclusion criteria

These CSF samples were obtained from clinical trials, including NCT03225651, NCT05307536, NCT02569775, NCT03123562, NCT02574923, NCT05472428, and previous reports^7,9,17–19^. Specifically, these clinical trials focused on assessing the safety and efficacy of intrathecal autologous BMMNC infusion in children with ASD, CP, and SB. In the process of intrathecal BMMNC infusion, it was required to remove a volume of CSF equivalent to the volume of infused BMMNC suspension to maintain intracranial pressure. Consequently, these CSF samples were collected for analysis in this study.

CSF samples from ASD patients were sourced from clinical trials NCT03225651 and NCT05307536^7^. Inclusion criteria for ASD patients were as follows: patients diagnosed with ASD according to the Diagnostic and Statistical Manual of Mental Disorders, Fifth Edition^44^, with Childhood Autism Rating Scale scores exceeding 37, both sexes, and aged between three and 11 years. Exclusion criteria included ASD patients with epilepsy, hydrocephalus with ventricular drain, coagulation disorders, allergy to anesthetic agents, severe health conditions (such as cancer or heart, lung, liver, or kidney failure), active infectious diseases, and patients with Rett syndrome or fragile X syndrome.

CSF samples from CP patients were obtained from clinical trials NCT02569775, NCT03123562, and NCT02574923^9,17,18^. Inclusion criteria for CP patients were: (i) patients with CP having a Gross Motor Function Classification System^45^ score ranging from level II to level V, both sexes, aged from one to 16 years; and (ii) a previous history of icterus during the neonatal period. Patients with coagulation disorder, serious illnesses such as cancer; failure of the heart, lungs, liver, or kidneys; or an active form of infection were excluded from this study.

CSF samples from SB patients were obtained from clinical trial NCT05472428^19^. Inclusion criteria for SB patients were patients diagnosed with lumbar spina bifida who underwent spinal cord close-up surgery and exhibited bowel disorders (constipation, fecal incontinence) as well as urinary dysfunction (urinary retention or leakage), both sexes, aged between one and 18 years old. Exclusion criteria comprised (i) vertebrae clefts in the chest, neck, and other spinal locations, (ii) coagulopathy, acute and chronic infection, (iii) kidney function disorder, liver failure, and (iv) complex cardiovascular diseases (including valvular heart disease, cardiomyopathy, arrhythmia, congenital heart disease, hypertrophy syndrome).

CSF samples were obtained from 26 ASD, 28 CP, and eight SB patients before autologous BMMNC infusion. In the case of ASD and CP, CSF samples were collected twice, one before the first autologous BMMNC infusion and again before the second infusion. The extraction procedure involved obtaining CSF from the space between two lumbar bones in the lower back, using an 18G needle. The collected volume of CSF matched the volume of the infused BMMNC suspension (3–5 mL). The samples were then transferred into 15 mL polypropylene tubes.

### Isolation and infusion of bone marrow mononuclear cells

Isolation and infusion of BMMNCs were detailed in clinical trials NCT03225651, NCT05307536, NCT02569775, NCT03123562, NCT02574923, NCT05472428, and previous reports^7,9,17–19^ from which the CSF samples were obtained for this study.

### CSF preparation

CSF samples were prepared in accordance with the BioMS guidelines^49^. Briefly, the obtained CSF were stored at 4 °C for a maximum of four hours or immediately processed. The CSF sample was first centrifuged at 400 × g for 10 min at 4 °C, and then the supernatant was harvested, aliquoted into polypropylene tubes, labeled with bar codes, and stored at − 80 °C until needed for subsequent analysis. The remaining pellet was resuspended in 100 µL PBS for cell count. Only non-blood contaminated CSF samples (red blood cell counts of < 500 per µL CSF) were considered for further analysis.

### Cytokine and growth factor analysis

Cytokines and GFs, including (APRIL, BAFF, BDNF, BMP-9, EGF, FGF-2, G-CSF, GDF-15, GM-CSF, HGF, IFN-γ, IL-1β, IL-2, IL-4, IL-5, IL-6, IL-10, IL-12p70, IL-17A, IL-21, IL-31, IL-33R, M-CSF, NGF-β, PDGF, TGF-β, TNF-α and VEGF), were selected based on previous reports showing their significant changes in biological liquids of the patients with ASD and CP (Supplemental Table 1). The concentration of these molecules in CSF was quantified by ProcartaPlex™ Multiplex Immunoassays (ThermoFisher, Massachusetts, US) using a Luminex® 100/200™ System. Frozen CSF samples were thawed and kept on ice, then processed following the manufacturer’s instructions. Briefly, 50 µL of Magnetic Beads solution was pre-coated to each well of a 96-well plate. The liquid was then discarded, and the wells were washed with 150 µL of Wash Buffer. Subsequently, 25 µL Universal Assay Buffer 1X was added to each well, followed by 25 µL of pre-prepared standards or samples into dedicated wells. The plate was then shaken at RT at 500 rpm for 30 min and incubated overnight at 4 °C. After 2 times of washing, 25 µL Detection Antibody Mixture 1X was added to each well and incubated for 30 min in the dark on a plate shaker at RT and shaken at 500 RPM. Next, the wells were washed twice before adding 50 µL Streptavidin-PE per well and incubated for another 30 min as described in the previous step. After washing, 50 µL of Amplification Reagent was added and incubated for 30 min at RT. After washing, Reading Buffer for luminescence detection was added and incubated for 5 min at room temperature at 500 rpm on a shaker before the reading step. The luminescent signal was detected using a Luminex™ 100/200™ system with xPONENT 3.1 software.

Data generated from the Luminex assay were subjected to the ProCartaPlex Analyst 1.0 Software for quantitative analysis. Calibration curves were constructed using a serial diluted standard range.

Each aliquot of CSF samples in this study was frozen and thawed only once.

### Statistical analysis

Statistical analysis was performed using GraphPad Prism 9. Data were presented as Mean ± SD or Mean ± SEM as indicated in the tables and figure legends. Groups were compared using two-way ANOVA, T-test or Wilcoxon Signed Rank Test as indicated in figure legends. Considered *p*-values were: ^ns^p > 0.05; *p < 0.05; **p < 0.01; *** *p* < 0.001; ****p < 0.0001.

### Ethics approval 

The study protocol was reviewed and approved by the Institutional Review Board of Vinmec International Hospital (Reference number 150117/2017/QĐ-VMEC) in accordance with Declaration of Helsinki.

### Patient consent

The study was explained in detail to the parents of the participants, and written informed consent forms were obtained before sample collection. Patients did not have to pay for their participation into the study. The authors stated that no identifiable images or identifiable data are presented in the manuscript.

### Supplementary Information

Supplementary Table 1.

## Data Availability

All data are available in the main text or the supplementary materials.
