# Supplementary material for: Inflammatory mediators drive neuroinflammation in autism spectrum disorder and cerebral palsy
Source: Sci Rep. 2023 Dec 18;13:22587. doi: 10.1038/s41598-023-49902-8 (PMC10730823; doi:10.1038/s41598-023-49902-8)
Supplement: Supplementary file 1 — Supplementary Table 1. [file 41598_2023_49902_MOESM1_ESM.docx]

**Supporting information**

**Supplemental table 1: Studies of cytokines and growth factors in ASD and CP patients**

| **Cytokines/ Growth factors** | **Published literatures** | |
| --- | --- | --- |
|  | **ASD** | **CP** |
| APRIL | Plasma anti-myelin basic protein (anti-MBP) auto-antibodies are significantly higher in ASD than healthy control children, suggesting antibody-secreting cells might be involved in the pathogenesis of ASD [1]**.** APRIL is required for the maintenance and survival of antibody secreting cells [2], thus it is important to examine whether APRIL is present in the CSF from ASD patients as this might reveal antibody-mediated neuroinflammation mechanisms in ASD |  |
| BAFF | - Plasma anti-myelin basic protein (anti-MBP) auto-antibodies are significantly higher in ASD than healthy control children, suggesting antibody-secreting cells might be involved in the pathogenesis of ASD [1]**.** As APRIL, BAFF is also important for the maintenance and survival of antibody-secreting cells [2], thus it is important to examine whether BAFF is present in the CSF from ASD patients since this might reveal antibody-mediated neuroinflammation mechanisms in ASD  - Serum BAFF has been shown to associate with ASD [3]  - BAFF is increased in the CSF of patients with myoclonus syndrome and it is associated with disease severity and neuroinflammation [4] |  |
| BDNF | Increased in ASD serum compared to healthy control [5-8] | Decreased in the ASD plasma compared to healthy control **[9]** |
| BMP-9 | BMP/BMPRIA signaling is involved in the differentiation of neural precursor cells into astrocytes, and subsequently astrocytes regulate the expression of VEGF for proper cerebrovascular angiogenesis which is important for the formation of the blood–brain-barrier [10]. There is increasing evidence showing that altered expression of genes associated with blood brain barrier was found in ASD brain and this change correlated with increased neuroinflammation in this disease [11, 12]. Thus, it is important to check for the level of BMP-9 in the CSF of ASD patients |  |
| EGF | Increased in serum of ASD compared to healthy control [13, 14] | - Increased in serum of CP compared to control [15]  - Increased in preterm infants with CP compared to control [15] |
| FGF-2 | Decreased in the serum of ASD compared to healthy control [16] |  |
| G-CSF | Decreased in plasma of ASD compared to siblings without ASD [17] | **Used in clinical trial NCT02866331 to treat CP patients [18]** |
| GDF-15 | Increased in patients with neurodegenerative disease compared to healthy people [19] and associated with brain disorders [20] | Increased in patients with neurodegenerative disease compared to healthy people [19] and associated with brain disorders [20] |
| GM-CSF | - Children with ASD exhibited significantly increased number of GM-CSF-expressing cells in the peripheral blood compared with healthy children [21]  - M2 cells secrete GM-CSF that possess neuroprotective function and induce CNS regeneration [22] | - Decreased in preterm infants with CP compared to control [15]  - M2 cells secrete GM-CSF that possess neuroprotective function and induce CNS regeneration [22] |
| HGF | Decreased in the serum of ASD Children with Severe Gastrointestinal Disease [23] |  |
| IFN-γ | - Children with ASD exhibited significantly increased number of IFN-γ-expressing cells in the peripheral blood compared to healthy children [21]  - Increased in ASD plasma compared to healthy control [24, 25] | Increased in the blood of CP patients compared to the healthy control [26] |
| IL-1β | - Increased in ASD plasma/serum compared to healthy control [27-30]  - Decreased in ASD serum compared to Ret syndrome [31] |  |
| IL-2 | Increased in ASD plasma compared to healthy control [32] | Decreased in preterm infants with CP compared to control [15] |
| IL-4 | Increased in the serum of ASD compared to healthy control [33-35] | Regulated proinflammatory cytokine responses [36] |
| IL-5 | Increased in ASD plasma compared to healthy control [28] | Increased in CP serum compared to control [15] |
| IL-6 | - - Children with ASD exhibited a significantly increased number of IL-6-expressing cells in the peripheral blood than healthy control [21]   - Increased in ASD plasma/serum compared to healthy control [27, 29, 33, 37, 38] | - Increased in the serum of moderate CP compared to mild CP [39]  - Increased in the serum of CP children compared to healthy control [40, 41] |
| IL-10 | - Decreased in the serum of ASD patients compared to healthy control [42, 43] | - Increased in the plasma of CP compared to healthy control [44] |
| IL-12p70 | Increased in ASD plasma compared to the healthy control [24, 28, 29] | Increased in CP serum compared to control [15] |
| IL-17A | Increased in ASD plasma/serum compared to the healthy control [28-30, 45] |  |
| IL-21 | Increased CXCR1^+^IL-21^+^ cells in the blood of ASD compared to those from healthy control [46] |  |
| IL-31 | - Increased CXCR1^+^IL-31^+^ cells in the blood of ASD compared to those from healthy control [46] |  |
| IL-33R | IL-33 is expressed by brain and spinal cord tissue and plays an important role in immune-mediated disease, suggesting potential roles in pathogenesis of neurological diseases [47]. Thus, it is important to examine the level of IL-33R in order to understand whether the IL-33/IL-33R signaling pathway participates to the ASD development |  |
| M-CSF | M-CSF promotes the generation of a new subset of tissue repair macrophage for traumatic brain injury recovery [48] | M-CSF promotes the generation of a new subset of tissue repair macrophage for traumatic brain injury recovery [48] |
| NGF-β | Increased in ASD plasma/serum compared to the control [1, 49-51] |  |
| PDGF | - Decreased in the serum of male children with ASD than those of healthy control [52]  - Increased in ASD children compared to the control children **[53]** |  |
| TGF-β | - - Decreased in the serum/plasma of ASD patients compared to healthy control [54-56] - - TGF-β has a neuroprotective function via inducing the differentiation of neural stem cells [57, 58] |  |

**References**

1. Mostafa, G.A., et al., *Plasma levels of nerve growth factor in Egyptian autistic children: Relation to hyperserotonemia and autoimmunity.* J Neuroimmunol, 2021. **358**: p. 577638.

2. Schneider, P., *The role of APRIL and BAFF in lymphocyte activation.* Current Opinion in Immunology, 2005. **17**(3): p. 282-289.

3. Smedler, E., et al., *Cerebrospinal fluid and serum protein markers in autism: A co-twin study.* J Neurochem, 2021. **158**(3): p. 798-806.

4. Pranzatelli, M.R., et al., *BAFF/APRIL system in pediatric OMS: relation to severity, neuroinflammation, and immunotherapy.* J Neuroinflammation, 2013. **10**: p. 10.

5. Miyazaki, K., et al., *Serum neurotrophin concentrations in autism and mental retardation: a pilot study.* Brain and Development, 2004. **26**(5): p. 292-295.

6. Barbosa, A.G., et al., *Assessment of BDNF serum levels as a diagnostic marker in children with autism spectrum disorder.* Sci Rep, 2020. **10**(1): p. 17348.

7. Wang, M., et al., *Increased serum levels of brain-derived neurotrophic factor in autism spectrum disorder.* Neuroreport, 2015. **26**(11): p. 638-41.

8. Ricci, S., et al., *Altered cytokine and BDNF levels in autism spectrum disorder.* Neurotox Res, 2013. **24**(4): p. 491-501.

9. Hansen, S.L., et al., *Suboptimal nutrition and low physical activity are observed together with reduced plasma brain-Derived neurotrophic factor (BDNF) concentration in children with severe cerebral palsy (CP).* Nutrients, 2019. **11**(3): p. 620.

10. Araya, R., et al., *BMP signaling through BMPRIA in astrocytes is essential for proper cerebral angiogenesis and formation of the blood–brain-barrier.* Molecular and Cellular Neuroscience, 2008. **38**(3): p. 417-430.

11. Fiorentino, M., et al., *Blood-brain barrier and intestinal epithelial barrier alterations in autism spectrum disorders.* Mol Autism, 2016. **7**: p. 49.

12. Srinivasjois, R., S. Rao, and S. Patole, *Probiotic supplementation in children with autism spectrum disorder.* Arch Dis Child, 2015. **100**(5): p. 505-6.

13. Tobiasova, Z., et al., *Risperidone-related improvement of irritability in children with autism is not associated with changes in serum of epidermal growth factor and interleukin-13.* J Child Adolesc Psychopharmacol, 2011. **21**(6): p. 555-64.

14. Pardo, C.A., et al., *Serum and cerebrospinal fluid immune mediators in children with autistic disorder: a longitudinal study.* Mol Autism, 2017. **8**: p. 1.

15. Kaukola, T., et al., *Cerebral palsy is characterized by protein mediators in cord serum.* Annals of Neurology, 2004. **55**(2): p. 186-194.

16. Esnafoglu, E. and S.N. Ayyildiz, *Decreased levels of serum fibroblast growth factor-2 in children with autism spectrum disorder.* Psychiatry Res, 2017. **257**: p. 79-83.

17. Manzardo, A.M., et al., *Plasma cytokine levels in children with autistic disorder and unrelated siblings.* Int J Dev Neurosci, 2012. **30**(2): p. 121-7.

18. Rah, W.J., et al., *Neuroregenerative potential of intravenous G-CSF and autologous peripheral blood stem cells in children with cerebral palsy: a randomized, double-blind, cross-over study.* J Transl Med, 2017. **15**(1): p. 16.

19. Xue, X.H., et al., *Diagnostic utility of GDF15 in neurodegenerative diseases: A systematic review and meta-analysis.* Brain Behav, 2022. **12**(2): p. e2502.

20. Jiang, W.-W., et al., *Emerging roles of growth differentiation factor‑15 in brain disorders (Review).* Exp Ther Med, 2021. **22**(5): p. 1270.

21. Ahmad, S.F., et al., *Involvement of CD45 cells in the development of autism spectrum disorder through dysregulation of granulocyte-macrophage colony-stimulating factor, key inflammatory cytokines, and transcription factors.* Int Immunopharmacol, 2020. **83**: p. 106466.

22. Elena R. Chernykh, et al., *The generation and properties of human M2-like macrophages: potential candidates for CNS repair?* Cellular Therapy and Transplantation, 2010. **2**(6).

23. Russo, A.J., et al., *Decreased Serum Hepatocyte Growth Factor (HGF) in Autistic Children with Severe Gastrointestinal Disease.* Biomark Insights, 2009. **4**: p. 181-90.

24. Singh, V.K., *Plasma increase of interleukin-12 and interferon-gamma. Pathological significance in autism.* Journal of Neuroimmunology, 1996. **66**(1): p. 143-145.

25. Tostes, M.H.F., et al., *Altered neurotrophin, neuropeptide, cytokines and nitric oxide levels in autism.* Pharmacopsychiatry, 2012. **45**(06): p. 241-243.

26. Grether, J.K., et al., *Interferons and cerebral palsy.* J Pediatr, 1999. **134**(3): p. 324-32.

27. Ashwood, P., et al., *Elevated plasma cytokines in autism spectrum disorders provide evidence of immune dysfunction and are associated with impaired behavioral outcome.* Brain Behav Immun, 2011. **25**(1): p. 40-5.

28. Suzuki, K., et al., *Plasma cytokine profiles in subjects with high-functioning autism spectrum disorders.* PLoS One, 2011. **6**(5): p. e20470.

29. Jácome, M.C.C., et al., *Peripheral inflammatory markers contributing to comorbidities in autism.* Behav Sci (Basel), 2016. **6**(4).

30. Xie, J., et al., *Immunological cytokine profiling identifies TNF-α as a key molecule dysregulated in autistic children.* Oncotarget, 2017. **8**(47).

31. Pecorelli, A., et al., *Cytokines profile and peripheral blood mononuclear cells morphology in Rett and autistic patients.* Cytokine, 2016. **77**: p. 180-188.

32. Singh, V.K., et al., *Changes of soluble interleukin-2, interleukin-2 receptor, T8 antigen, and interleukin-1 in the serum of autistic children.* Clin Immunol Immunopathol, 1991. **61**(3): p. 448-55.

33. Kordulewska, N.K., et al., *Serum cytokine levels in children with spectrum autism disorder: Differences in pro- and anti-inflammatory balance.* J Neuroimmunol, 2019. **337**: p. 577066.

34. Zhao, H., et al., *Association of Peripheral Blood Levels of Cytokines With Autism Spectrum Disorder: A Meta-Analysis.* Front Psychiatry, 2021. **12**: p. 670200.

35. Krakowiak, P., et al., *Neonatal cytokine profiles associated with autism spectrum disorder.* Biol Psychiatry, 2017. **81**(5): p. 442-451.

36. Dinarello, C.A., *Proinflammatory cytokines.* Chest, 2000. **118**(2): p. 503-508.

37. Tsilioni, I., et al., *Children with autism spectrum disorders, who improved with a luteolin-containing dietary formulation, show reduced serum levels of TNF and IL-6.* Transl Psychiatry, 2015. **5**(9): p. e647.

38. Shaker, N., et al., *Serum levels of S100b, interleukin-6 and anti-transglutaminase Ii IgA as immune markers in a sample of egyptian children with autistic spectrum disorders.* Autism-Open Access, 2016. **6**: p. 1-8.

39. Chalak, L.F., et al., *Biomarkers for severity of neonatal hypoxic-ischemic encephalopathy and outcomes in newborns receiving hypothermia therapy.* J Pediatr, 2014. **164**(3): p. 468-74.e1.

40. Bi, D., et al., *The association between sex-related interleukin-6 gene polymorphisms and the risk for cerebral palsy.* Journal of neuroinflammation, 2014. **11**(1): p. 1-12.

41. Pingel, J., et al., *Systemic inflammatory markers in individuals with cerebral palsy.* European Journal of Inflammation, 2019. **17**: p. 2058739218823474.

42. Bryn, V., et al., *Cytokine profile in autism spectrum disorders in children.* Journal of molecular neuroscience : MN, 2017. **61**(1): p. 1-7.

43. Saghazadeh, A., et al., *Anti-inflammatory cytokines in autism spectrum disorders: A systematic review and meta-analysis.* Cytokine, 2019. **123**: p. 154740.

44. Xia, L., et al., *Combined Analysis of Interleukin-10 Gene Polymorphisms and Protein Expression in Children With Cerebral Palsy.* Front Neurol, 2018. **9**: p. 182.

45. Al-Ayadhi, L.Y. and G.A. Mostafa, *Elevated serum levels of interleukin-17A in children with autism.* J Neuroinflammation, 2012. **9**: p. 158.

46. Ahmad, S.F., et al., *Upregulation of interleukin (IL)-31, a cytokine producing CXCR1 peripheral immune cells, contributes to the immune abnormalities of autism spectrum disorder.* J Neuroimmunol, 2020. **349**: p. 577430.

47. Fairlie-Clarke, K., et al., *Expression and Function of IL-33/ST2 Axis in the Central Nervous System Under Normal and Diseased Conditions.* Front Immunol, 2018. **9**: p. 2596.

48. Li, Z., et al., *M-CSF, IL-6, and TGF-beta promote generation of a new subset of tissue repair macrophage for traumatic brain injury recovery.* Sci Adv, 2021. **7**(11).

49. Mostafa, G.A., et al., *Plasma levels of nerve growth factor in Egyptian autistic children: Relation to hyperserotonemia and autoimmunity.* Journal of Neuroimmunology, 2021. **358**: p. 577638.

50. Dincel, N., et al., *Serum nerve growth factor levels in autistic children in Turkish population: a preliminary study.* Indian J Med Res, 2013. **138**(6): p. 900-3.

51. Liu, S.H., et al., *Peripheral blood neurotrophic factor levels in children with autism spectrum disorder: a meta-analysis.* Sci Rep, 2021. **11**(1): p. 15.

52. Kajizuka, M., et al., *Serum levels of platelet-derived growth factor BB homodimers are increased in male children with autism.* Prog Neuropsychopharmacol Biol Psychiatry, 2010. **34**(1): p. 154-8.

53. Zakareia, F.A., L.Y. Al-Ayadhi, and A.A. Al-Drees, *Study of dual angiogenic/neurogenic growth factors among Saudi autistic children and their correlation with the severity of this disorder.* Neurosciences (Riyadh), 2012. **17**(3): p. 213-8.

54. Ashwood, P., et al., *Decreased transforming growth factor beta1 in autism: a potential link between immune dysregulation and impairment in clinical behavioral outcomes.* J Neuroimmunol, 2008. **204**(1-2): p. 149-53.

55. Ohja, K., et al., *Neuroimmunologic and Neurotrophic Interactions in Autism Spectrum Disorders: Relationship to Neuroinflammation.* Neuromolecular Med, 2018. **20**(2): p. 161-173.

56. Khakzad, M.R., et al., *Transforming growth factor beta 1 869T/C and 915G/C polymorphisms and risk of autism spectrum disorders.* Rep Biochem Mol Biol, 2015. **3**(2): p. 82-8.

57. Dobolyi, A., et al., *The neuroprotective functions of transforming growth factor beta proteins.* Int J Mol Sci, 2012. **13**(7): p. 8219-58.

58. Rubio, J.A., *Plasma growth factors in neuronal regeneration.* Austin J Clin Neurol, 2017. **4**(3).
